# Arrhythmic Risk Assessment of Hypokalaemia Using Human Pluripotent Stem Cell-Derived Cardiac Anisotropic Sheets

**DOI:** 10.3389/fcell.2021.681665

**Published:** 2021-12-06

**Authors:** Bimal Gurung, Gary Tse, Wendy Keung, Ronald A. Li, Wing Tak Wong

**Affiliations:** ^1^ School of Life Sciences, The Chinese University of Hong Kong, Hong Kong SAR, China; ^2^ Cardiac Electrophysiology Unit, Cardiovascular Analytics Group, China-UK Collaboration, Hong Kong SAR, China; ^3^ Kent and Medway Medical School, Canterbury, Kent, United Kingdom; ^4^ Tianjin Key Laboratory of Ionic-Molecular Function of Cardiovascular Disease, Department of Cardiology, Tianjin Institute of Cardiology, Second Hospital of Tianjin Medical University, Tianjin, China; ^5^ Novoheart, Irvine, CA, United States; ^6^ Dr. Li Dak-Sum Research Centre, HKU-Karolinska Institutet Collaboration in Regenerative Medicine, The University of Hong Kong, Hong Kong SAR, China; ^7^ Shenzhen Research Institute, The Chinese University of Hong Kong, Shenzhen, China; ^8^ State Key Laboratory of Agrobiotechnology, The Chinese University of Hong Kong, Hong Kong SAR, China

**Keywords:** stem cells, ventricular arrhythmias, hypokalaemia, optical mapping, action potential

## Abstract

**Introduction:** Hypokalaemia, defined as an extracellular concentration of K^+^ below 3.5 mM, can cause cardiac arrhythmias by triggered or re-entrant mechanisms. Whilst these effects have been reported in animal and human stem cell-based models, to date there has been no investigation in more complex structures such as the human ventricular cardiac anisotropic sheet (hvCAS). Here, we investigated arrhythmogenicity, electrophysiological, and calcium transient (CaT) changes induced by hypokalaemia using this bioengineered platform.

**Methods:** An optical mapping technique was applied on hvCAS derived from human pluripotent stem cells to visualize electrophysiological and CaT changes under normokalaemic (5 mM KCl) and hypokalaemic (3 mM KCl) conditions.

**Results:** Hypokalaemia significantly increased the proportion of preparations showing spontaneous arrhythmias from 0/14 to 7/14 (Fisher’s exact test, *p* = 0.003). Hypokalaemia reduced longitudinal conduction velocity (CV) from 7.81 to 7.18 cm⋅s^−1^ (n = 9, 7; *p* = 0.036), transverse CV from 5.72 to 4.69 cm⋅s^−1^ (n = 12, 11; *p* = 0.030), prolonged action potential at 90% repolarization (APD_90_) from 83.46 to 97.45 ms (n = 13, 15; *p* < 0.001), increased action potential amplitude from 0.888 to 1.195 ΔF (n = 12, 14; *p* < 0.001) and CaT amplitude from 0.76 to 1.37 ΔF (n = 12, 13; *p* < 0.001), and shortened effective refractory periods from 242 to 165 ms (n = 12, 13; *p* < 0.001).

**Conclusion:** Hypokalaemia exerts pro-arrhythmic effects on hvCAS, which are associated with alterations in CV, repolarization, refractoriness, and calcium handling. These preparations provide a useful platform for investigating electrophysiological substrates and for conducting arrhythmia screening.

## Introduction

Hypokalaemia is defined as an extracellular concentration of K^+^ below the normal range of 3.5–5.3 mM (Macdonald and Struthers, 2004; Unwin et al., 2011). It is a common biochemical abnormality in cardiac patients that may represent a side effect of diuretic therapy or result from endogenous activation of the renin-angiotensin system and high adrenergic tone. Transient hypokalaemia is also induced by intense exercise during recovery (Atanasovska et al., 2018). It is a risk factor for ventricular arrhythmias that can predispose patients to sudden cardiac death (Osadchii, 2010; Chen et al., 2021). The mortality rate of hospitalized patients with hypokalaemia is tenfold higher than in the generalized hospital population, illustrating its potentially life-threatening consequences (Paltiel et al., 2001). Low levels of K^+^ are replaced with supplements through oral or intravenous routes.

Hypokalaemia can induce several electrophysiological abnormalities, such as delayed ventricular repolarization, which in turn predisposes patients to triggered activity through the development of early afterdepolarizations (EADs) and re-entrant arrhythmias by increasing the dispersion of repolarization (Schulman and Narins, 1990; Macdonald and Struthers, 2004; Osadchii, 2010). Its pro-arrhythmic effects and mechanisms have been demonstrated in previous studies in animal and human stem cell-based models (Tse et al., 2016b; Kuusela et al., 2017). However, to date, there have been no investigations into more complex structures such as the human ventricular cardiac anisotropic sheet (hvCAS). hvCAS is a micro-patterned substrate that is specifically designed to reproduce the anisotropy of native human ventricles and allows for direct visualization of arrhythmic spiral re-entry (Chen et al., 2011; Luna et al., 2011; Wang et al., 2013; Shum et al., 2017; Wong et al., 2019). In this study, we investigated the electrophysiological and calcium transient (CaT) changes induced by hypokalaemia using this bioengineered platform (Wang et al., 2013; Shum et al., 2017). An optical mapping technique was applied on hvCAS derived from human pluripotent stem cells to investigate electrophysiological and CaT changes on normokalaemic (5 mM KCl) and hypokalaemic (3 mM KCl) preparations.

## Materials and Methods

This study has been approved by the University of Hong Kong/Hospital Authority Hong Kong West Cluster Institutional Review Board. Informed consent was given by the subject.

### Reprogramming and Cell Culture

A peripheral blood sample was obtained from a normal healthy adult person. CD34^+^ cells were isolated from peripheral blood mononuclear cells (>95% purity) by using human CD34 Microbead Kit (130-046-702; Miltenyi Biotec) and MACS system (130-042-201; 130-098-308; 130-042-303; Miltenyi Biotec). They were expanded for 3 days in StemSpan™ H3000 (Stemcell Technologies) with the cytokine cocktail CC100 (Stemcell Technologies).

Nucleofection was performed on them with the non-integrated episomal vectors pCXLE-hOCT3/4-shp53, pCXLE-hSK, and pCXLE-hUL (Okita et al., 2011) using the Human CD34 Cell Nucleofector™ kit (Amaxa) according to the manufacturer’s protocol for reprogramming.

Cells were then seeded to Geltrex (A1413302; Gibco)-coated plates in E8 medium (Life Technologies). After a few days, morphological changes were observed and human induced pluripotent stem (iPS) cell colonies typically started to appear on day 7. Tra-1-60 enrichment was performed on day 13 after nucleofection.

Human embryonic stem cells (hES2) (ESI, NIH code ES02) (passages 35–80) and human iPS cell (passages 50–90) lines were maintained in feeder- and serum-free conditions in mTeSR1 medium (STEMCELL Technologies) on Matrigel™ (BD Biosciences) and E8 medium on Geltrex respectively at 37°C in 5% CO_2_ and 21% O_2_ in a humidified normoxic environment and cell passaging was performed every 3, 4 days at 70–80% confluence using Accutase (A11105; Gibco). A tissue culture-treated six-well plate (3,506; Coster) was enrolled. The cells were negative in the mycoplasma test.

### Human iPS Cells Validation

The human iPS cell colonies were treated with 4% paraformaldehyde in phosphate-buffered saline (PBS) for 15 min for fixation and then permeabilized by treating with 0.1% Triton X-100 for 15 min. They were then washed with PBS three times. Staining with anti-OCT4, SSEA-4, and Tra-1-81 for 2 h at room temperature were performed on the fixed samples. They were then treated with flurochrome-conjugated goat anti-rabbit or anti-mouse secondary antibodies for 1 h. For the pluripotency test, human iPS cells were differentiated to form Embroid Bodies (EB). The EB medium used was DMEM/F12 (Life Technologies) with 20% Knockout Serum Replacement (Life Technologies), 2 mM nonessential amino acids, 2 mM l-glutamine, and 0.1 mM β-mercaptoethanol. EBs were stained at day 15 for three germ layer markers (ie., Tuji, SMA, and AFP). Karyotyping was performed as described by Bates (Bates, 2011).

### Directed Cardiomyocyte Differentiation and Characterization

Human embryonic stem (ES) and iPS cells were digested into single-cells by Accutase and seeded onto Matrigel-coated and Geltrex-coated plates respectively at ∼ 3 × 10^5^ cells/10 cm^2^. An established directed cardiomyocyte differentiation protocol was used (Weng et al., 2014). ES cells were put in mTeSR1 medium and iPS cells were put in E8 medium for 4 days until ∼ 80–90% confluence. They were digested into single-cells by Accutase and transferred to ultralow-attachment six-well plates (Corning) for non-adherent culture in mTeSR1 medium (for ES cells) and E8 medium (for iPS cells), with Matrigel (40 μg/ml) with BMP4 (1 ng/ml; Invitrogen) and Rho kinase inhibitor (RI) (10 μM; R&D) under hypoxic conditions with 5% O_2_. This was termed Day (D) 0 as it was the initiation of cardiomyocyte differentiation. On D1 (24 h later), the culture was changed to StemPro34 SFM (Invitrogen) with ascorbic acid (AA, 50 μg/ml; Sigma), 2 mM GlutaMAX-1 (Invitrogen), BMP4 (10 ng/ml), and human recombinant activin-A (10 ng/ml; Invitrogen). On D4, the culture was changed to StemPro34 SFM + AA, 2 mM GlutaMAX-1, IWR-1, a Wnt inhibitor (5 μM; Enzo Life Sciences). Contracting clusters could be observed on D8 or later. On D8, the culture was changed to StemPro34 SFM + AA, 2 mM GlutaMAX-1, and transferred to a normoxic environment. From D8 onwards, cells were maintained in StemPro34 SFM + AA, 2 mM GlutaMAX-1, and replenished every 3–4 days for further characterization and downstream functional assays.

Flow cytometry analysis was performed on differentiated cells collected at D16–18 to quantify cTnT^+^ cells resembling cardiomyocyte yield.

### Fabrication of hvCAS

hvCAS substrates were fabricated according to a published protocol (Shum et al., 2017). The mold was obtained from Ming Wai Lau Centre for Reparative Medicine, Karolinska Institutet of groove dimensions 15 µM (R) × 5 µM (D) × 5 µM (W). Each substrate was 15 mm in diameter. Polystyrene shrink films (Clear Shrink Dinks) were hot-embossed at 180°C for 30 min. They were then treated with ultraviolet ozone (UVO) for 8 min (Jetlight UVO). Finally, they were submerged in 70% ethanol and kept under UV light for at least 15 min for sterilization. The substrates were coated with Matrigel and suctioning was performed for at least 15 min to remove air bubbles. The substrates were kept in a four-well plate (6900A07; Thermo Scientific Nunc) using vaseline.

**FIGURE 1:**
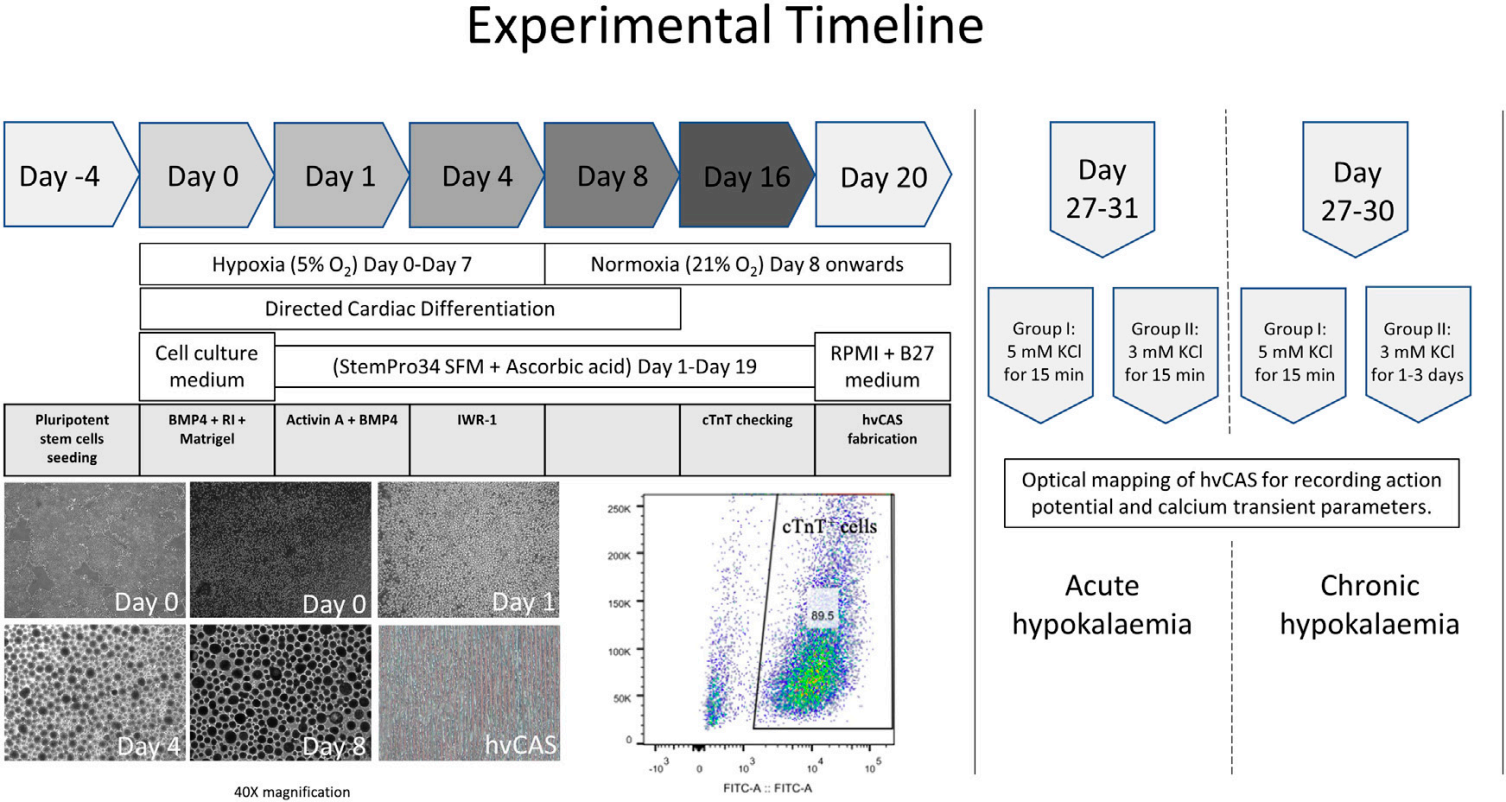
Experimental timeline from human pluripotent stem cell seeding, their directed cardiac differentiation, and hvCAS fabrication to an action potential and calcium transient data acquisition. The photos are of human embryonic stem cells (hES2), which show the cells’ morphology at different time points of directed cardiac differentiation. The photos are taken at 40X magnification. Cardiomyocyte yield from hES2 shows 89.5% cTnT^+^ cells, which were then used for downstream experiments.

Cardiomyocyte clusters (cTnT^+^ cells >60%) were digested into single-cells on D18-20 by treatment with 0.025% Trypsin-EDTA, phenol red (Gibco, Cat. No. 25300062, 500 ml, stored at –20°C) (4 ml per six-well plate) in PBS for 12 min at 37°C in a water bath with shaking at 90 rpm, which was then blocked by H1 medium with 20% Fetal Bovine Serum using twice the volume of 0.025% Trypsin-EDTA used. H1 medium with 20% Fetal Bovine Serum (FBS) medium was prepared by mixing 20% of FBS (Gibco, Cat. No. 1027016, 500 ml, stored at –20°C) in Dulbecco’s Modified Eagle Medium (DMEM) (High Glucose) (Gibco, Cat. No. 11965118, 500 ml, stored at 4°C) with Penicillin-Streptomycin (P/S)(1X) (Gibco, Cat. No. 15140122, 100 ml, stored at –20°C), GlutaMAX (1X) (Gibco, Cat. No. 35050061, 100 ml, stored at room temperature) and MEM Non-Essential Amino Acids (NEAA) (1X) (Gibco, Cat. No. 11140-035, 100 ml, stored at 4°C). The solution was then filtered through a 0.22 µM PES membrane. The digested cells were passed through 40 μM cell strainer (BD Biosciences). Cell seeding on the Matrigel-coated polystyrene substrate was done at 250 K cm^−2^ termed day 0 of hvCAS. The hvCAS were maintained on high glucose DMEM-based medium containing heat-inactivated 10% FBS, GlutaMAX (1X), MEM NEAA (1X) for 2 days at 37°C in 5% CO_2_ in a humidified normoxic environment. The medium was changed to RPMI 1640 medium (72,400; Gibco) with B-27 (17,504; Gibco) with P/S (1X) on the third day of seeding and replenished every second day. Optical mapping was performed from D7–D11 post hvCAS fabrication for electrophysiological examination after the formation of intercellular electrical junctions. The experimental timeline from cell culture to data acquisition is shown in Figure 1.

### Optical Mapping of hvCAS

Fresh normal Tyrode’s solution consisting of, in × 10^–3^ M, 140 NaCl, 5 KCl, 1 MgCl_2_, 1 CaCl_2_, 10 d-glucose, and 10 4-(2-hydroxyethyl)-1-piperazineethanesulfonic acid at pH 7.4 were prepared. For hypokalaemia, the freshly prepared Tyrode’s solution contained 3 × 10^–3^ M KCl whereas the other composition remained the same. The solution was consumed for up to 7 days.

hvCAS preparations were loaded with RH237 (5 × 10^–6^ M; Lot: 1,890,562; Life Technologies, membrane voltage sensitive dye), Rhod-2 AM (10 × 10^–6^ M; R1245MP; Thermofisher, calcium dye), and Pluronic F-127 (0.04%; P-3000MP; Life Technologies) for 20 min at 37°C in normal Tyrode’s solution (5 mM KCl for normokalaemic hvCAS) and hypokalaemic Tyrode solution (3 mM KCl for hypokalaemic hvCAS), followed by washing once with PBS and incubation for 10 min in Blebbistatin (50 × 10^–6^ M, B0560; Sigma-Aldrich) containing normal Tyrode’s solution and hypokalaemic Tyrode’s solution for the two subsets to minimize potential motion artifacts during optical mapping.

hvCASs were washed once with PBS and changed to normal Tyrode’s solution (for normokalaemia hvCAS) and hypokalaemic Tyrode’s solution (for hypokalaemia hvCAS) and maintained at 35–37°C using a culture dish incubator (Warners Instruments) for optical mapping. High-resolution optical mapping was performed using a MiCAM05 (SciMedia, CA, United States) for Action Potential (AP) and CaT parameters. The field of view was 17.6 mm by 17.6 mm obtained by using 1× objective and a 1× condensing lens. The input light source was a high-powered LED illumination system (LEX2, SciMedia, CA, United States) filtered by a 515 ± 35 nm band-pass excitation filter, and the light was split into two using a splitter. One was passed through a >700 nm long-pass emission filter for RH237 (voltage sensitive dye) and the other through a 590 nm band-pass emission filter for Rhod2-AM (calcium sensitive dye). Data acquisition was done at a sampling rate of 5 ms/frame and BVAna software (SciMedia) was used for data analysis followed by clampfit (Axon Instruments). A programmable electrical stimulator (Master9; AMPI, Israel) was used for pacing (10 V, 10 ms pulse duration) through a unipolar point-stimulation electrode (Harvard Apparatus, MA, United States). The point-stimulation electrode was positioned perpendicular to the hvCAS surface at its center. Optical mapping was performed once per sample and unpaired.

### Pacing Protocol for hvCAS

1) Steady state pacing was eight trains from 0.5 to 3 Hz (i.e., 30-180 bpm) at an increment of 0.5 Hz unless there was a loss of 1:1 capture. If they captured all the frequencies without arrhythmia, they were subjected to Programmed Electrical Stimulation (PES).

2) PES used eight trains of 1.5 Hz (S1) followed by a premature trigger (S2), where S1-S2 intervals were gradually shortened to find the Effective Refractory Period (ERP). The starting S1-S2 interval was 550 ms, which was then consecutively shortened in steps by 50 ms until capture had failed. S1-S2 interval was then increased by 20 ms, and then by 2 ms decrements until the ERP was achieved, which is the maximum S1-S2 interval that failed to lead to AP propagation (Figure 5C).

### hvCAS Subsets

Acute hypokalaemia: Optical mapping was performed on hvCAS for simultaneously measuring AP and CaT in normokalaemic and hypokalaemic conditions. All the hvCAS were maintained in the normokalaemic medium until optical mapping day (D7-D11 post hvCAS fabrication). On optical mapping day, normal Tyrode’s solution was used for one of the subsets (normokalaemia) and hypokalaemic Tyrode’s solution was used for another subset (hypokalaemia). The hvCAS were in respective Tyrode’s solution from dye loading to data acquisition (30 min to <60 min). The unpaired test was chosen as repeated measurement, which led to a significant decrease in the signal to noise ratio.

Chronic hypokalaemia: The fabricated hvCAS were divided into two subsets (Subset I: control, Subset II: experimental). Both groups were kept at normokalaemic condition until D7 post hvCAS fabrication. From D7–D10, optical mapping was performed for AP and CaT in which subset I was in normakalaemic condition throughout the experiment, whereas subset II was changed to hypokalaemic condition on D7–D10.

### AP and CaT Parameters

The following parameters were obtained from the experimental records:

1) Activation Latency or time-to-peak: The time interval from the start of the stimulus to the peak of the AP or CaT;
2) Amplitude: The height of the peak;
3) AP Duration (APD_x_) and CaT Duration (CaTD_x_): The time interval from the peak of the AP or CaT at x = 30, 50, 70, and 90% repolarization or decay respectively;
4) ERP: The maximum S1-S2 interval that failed to elicit an AP;
5) Upstroke Velocity: The velocity at which the fluorescence reaches the peak from baseline;
6) Conduction Velocity (CV): The velocity at which AP propagates between two points;
7) Anisotropic Ratio (AR): The ratio of longitudinal CV to transverse CV.

### Statistical Analysis

Prism (Version 7.0) and Microsoft Excel (Version 16.31) were used for statistical analysis. Values are in the median with lower and upper 95% Confidence Intervals. Different experimental groups were compared by Mann Whitney Test. Fisher’s exact test was used to analyze spiral induction. *p* < 0.05 was considered statistically significant. Data analysis was performed for 1.5 Hz electrical stimulation recordings.

## Results

### Acute Hypokalaemia Leads to Higher AP and CaT Amplitudes, and Longer APD and CaTD

**FIGURE 2:**
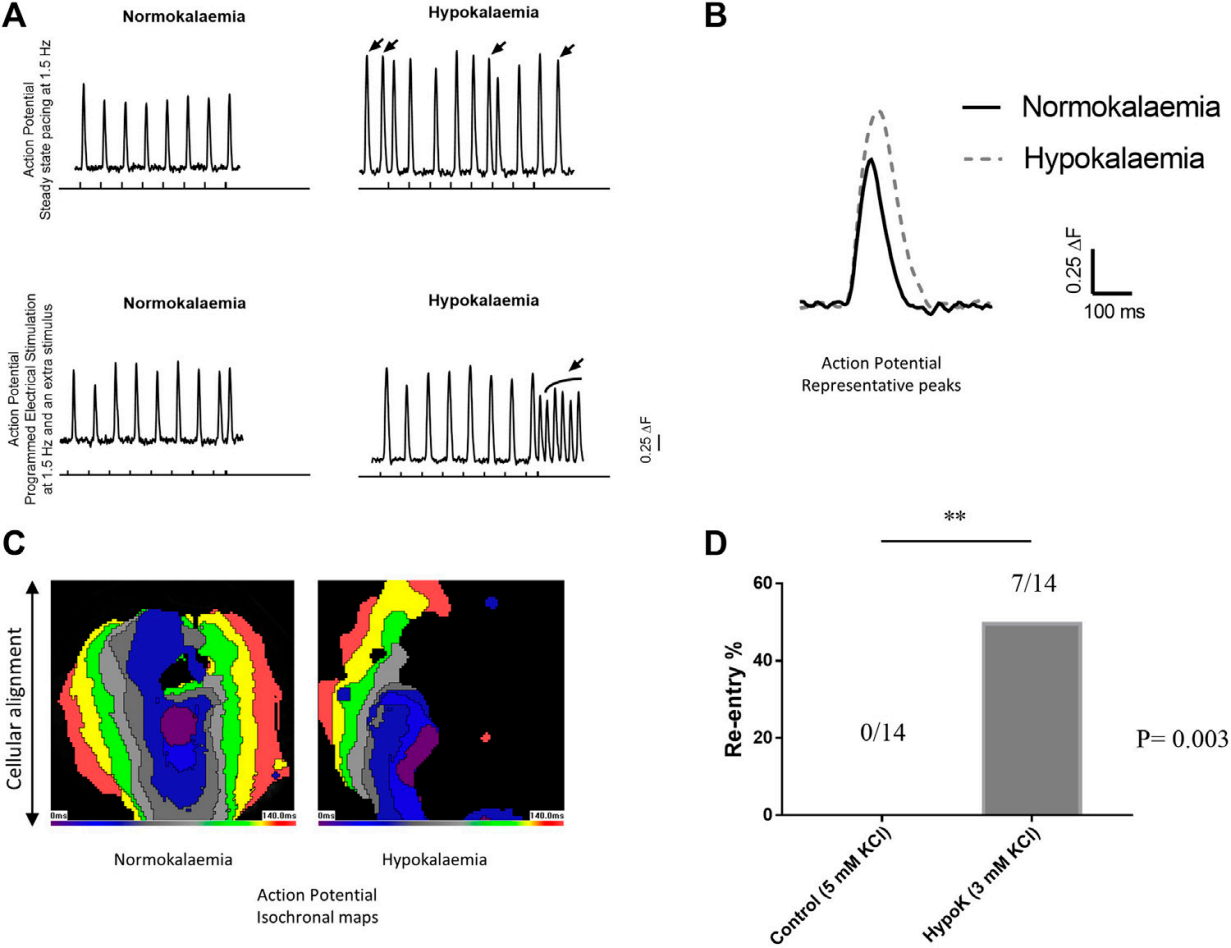
**(A)** Upper panel shows the steady state pacing at 1.5 Hz and the bottom panel shows programmed electrical stimulation. The perpendicular lines along the x-axis are the pacings from the external stimulator. Arrows over the peaks show premature action potentials (APs). **(B)** Representative AP tracings of normokalaemic and hypokalaemic samples. **(C)** Isochronal maps of the normokalaemic sample **(left panel)** with normal AP propagation from the center towards the edges and the hypokalaemic sample **(right panel)** showing spiral AP propagation circulating the center. **(D)** Re-entry percentages of normokalaemic and hypokalaemic samples.

Arrhythmogenicity and its relationship to AP and CaT activation and recovery properties were examined under normokalaemic and hypokalaemic conditions in hvCAS using optical mapping. During regular steady-state pacing, reproducible AP waveforms could be observed under normokalaemic conditions. By contrast, acute hypokalaemia led to the generation of premature APs that likely reflected triggered activity. Moreover, an S1S2 protocol, which delivered a successively premature S2 extra-stimulus after a regular train of S1 stimuli, was used to provoke arrhythmogenesis. With this protocol, an increase in the proportion of preparations exhibiting spiral wave re-entry was observed during hypokalaemia (hypokalaemia = 7/14, 50%; normokalaemia = 0/14, 0%; asterisks, *p* = 0.003 (Fisher’s exact test); Figure 2).

**FIGURE 3:**
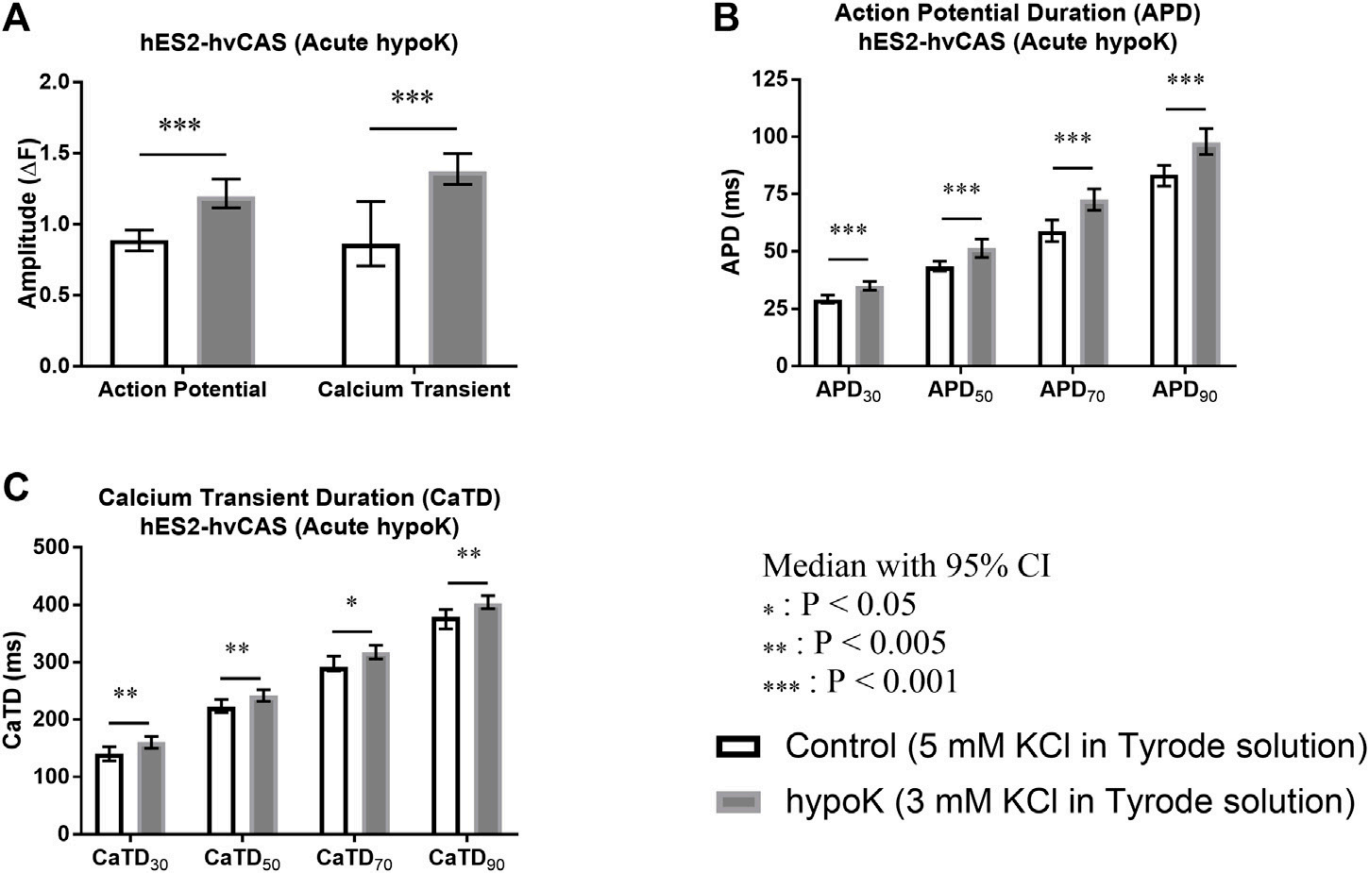
Human embryonic stem cell (hES2)-derived human ventricular cardiac anisotropic sheet (hvCAS) paced at 1.5 Hz during acute hypokalaemic (hypoK) experiment where the treatment was for 15 min before data acquisition. Number of samples is represented by n where the first number refers to control (5 mM KCl in Tyrode solution) samples and the second number refers to hypoK (3 mM KCl in Tyrode solution) samples. **(A)** Action Potential (AP) Amplitude (*n* = 12, 14) and Calcium Transient (CaT) Amplitude (*n* = 12, 13). ΔF represents the change in fluorescence intensity. **(B)** AP Duration (APD) at 30, 50, 70 and 90% fluorescence decay intervals from the peak fluorescence intensity represented by APD_30_, APD_50_, APD_70_ and APD_90_. (*n* = 13, 15). **(C)** CaT Duration (CaTD) at 30, 50, 70 and 90% fluorescence decay intervals from the peak fluorescence intensity represented by CaTD_30_, CaTD_50_, CaTD_70_ and CaTD_90_. (*n* = 11, 10). Amplitude of AP and CaT increased in hypokalaemic condition (asterisks, *p* < 0.001) and also for the APD and CaTD at 30, 50, 70 and 90% decay (asterisks, *p* < 0.001 for APD and *p* < 0.005, *p* < 0.05 for CaTD).

Acute hypokalaemia significantly increased AP amplitude (*p* < 0.001), and CaT amplitude (*p* < 0.001; Figure 3A). It also produced AP prolongation, as reflected by increases in APD_30_ (*p* < 0.001), APD_50_ (*p* < 0.001), APD_70_ (*p* < 0.001) and APD_90_ (*p* < 0.001; Figure 3B). CaTs were similarly prolonged during hypokalaemia, as reflected by higher CaTD. Thus, hyokalaemia increased CaTD_30_ (*p* = 0.001), CaTD_50_ (*p* = 0.003), CaTD_70_ (*p* = 0.009) and CaTD_90_ (*p* = 0.002; Figure 3C).

### Acute Hypokalaemia Prolongs AP and CaT Time-To-Peak, Increases AP and CaT Upstroke Velocities

**FIGURE 4:**
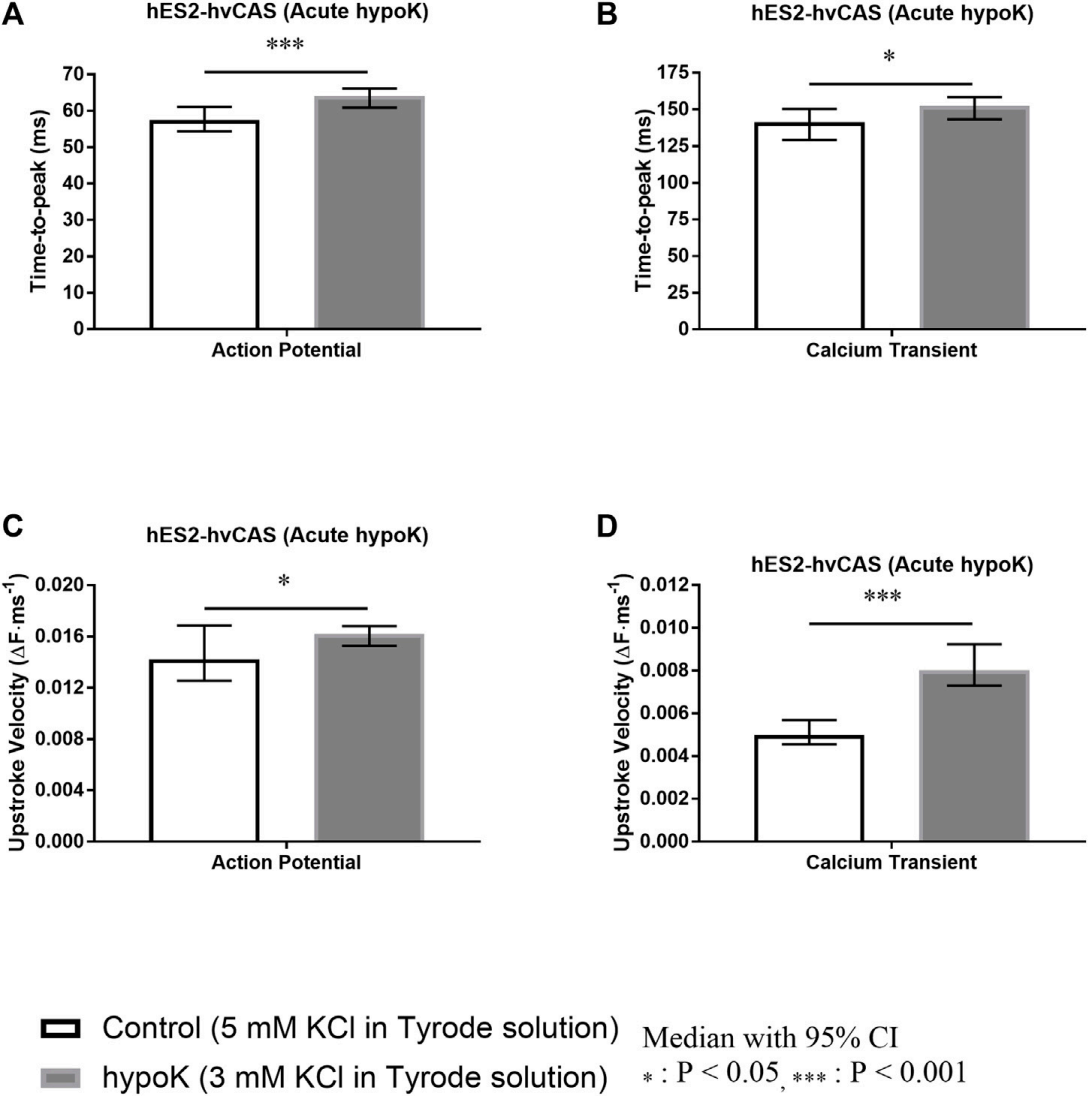
Human embryonic stem cell (hES2)-derived human ventricular cardiac anisotropic sheet (hvCAS) paced at 1.5 Hz during acute hypokalaemic (hypoK) experiment where the treatment was for 15 min before data acquisition. Number of samples is represented by n where the first number refers to control (5 mM KCl in Tyrode solution) samples and the second number refers to hypoK (3 mM KCl in Tyrode solution) samples. **(A)** Action Potential (AP) Time-to-peak. (*n* = 14, 17). **(B)** Calcium Transient (CaT) Time-to-peak. (*n* = 12, 11). **(C)** AP Upstroke Velocity measured by the change in fluorescence intensity per millisecond. ΔF represents the change in fluorescence intensity. (*n* = 9, 10). **(D)** CaT Upstroke Velocity measured by the change in fluorescence intensity per millisecond. ΔF represents the change in fluorescence intensity. (*n* = 11, 10) Time-to-peak of AP and CaT increased in hypokalaemic condition (asterisks, *p* < 0.001, *p* < 0.05 respectively). Similarly Upstroke Velocity of AP and CaT increased in hypokalaemic condition (asterisks, *p* < 0.05, *p* < 0.001 respectively).

The AP Time-to-peak was significantly increased during hypokalaemia (*p* < 0.001; Figure 4A). CaT Time-to-peak was also significantly altered in hypokalaemia (*p* = 0.045; Figure 4B).

Hypokalaemia increased AP upstroke velocity (*p* = 0.023; Figure 4C) and CaT upstroke velocity (*p* < 0.001; Figure 4D).

The AP and CaT values are summarized in Tables 1, 2 respectively.

**TABLE 1:** hES2-hvCAS Action Potential (AP) Parameters in acute hypokalaemia. Data analysis was performed for 1.5 Hz electrical stimulation recordings.

Parameters			Sample	Median	Lower 95% CI	Upper 95% CI	N	*p* Value
(a) Amplitude (ΔF represents the change in fluorescence intensity)			NormoK	0.888	0.81	0.958	12	*** < 0.001
			hypoK	1.19	1.12	1.32	14	
(b) AP Duration (APDx) (Time duration in millisecond from the peak fluorescence intensity to the corresponding fluorescence decay intervals)	x							
	30%		NormoK	29.1	27.6	31	13	*** < 0.001
			hypoK	34.9	33	37	15	
	50%		NormoK	43.4	41.4	45.8	13	*** < 0.001
			hypoK	51.5	47.4	55.3	15	
	70%		NormoK	59	54.4	63.7	13	*** < 0.001
			hypoK	72.6	68	77.2	15	
	90%		NormoK	83.5	78.5	87.5	13	*** < 0.001
			hypoK	97.4	92.2	104	15	
(c) Time-to-peak/Activation Latency (Time duration in millisecond from the start of the rise of the fluorescence intensity to the peak fluorescence intensity)			NormoK	57.4	54.4	61	14	*** < 0.001
			hypoK	64.1	60.9	66.1	17	
(d) Upstroke Velocity (ΔF/millisecond; ΔF represents the change in fluorescence intensity)			NormoK	0.0142	0.0126	0.0169	9	* = 0.023
			hypoK	0.0162	0.0153	0.0168	10	
(e) Conduction Velocity (CV) (cm·s^−1^; AP propagation between two points)	Longitudinal (L) CV		NormoK	7.81	7.01	10.6	9	* = 0.036
			hypoK	7.18	4.99	7.62	7	
	Transverse (T) CV		NormoK	5.72	4.83	7.02	12	* = 0.030
			hypoK	4.69	3.93	6.34	11	
(f) Anisotropic Ratio (Ratio of LCV to TCV)			NormoK	1.47	1.14	1.77	9	ns = 0.444
			hypoK	1.38	1.11	2.09	8	
(g) Effective Refractory Period (millisecond)			NormoK	242	200	275	12	*** < 0.001
			hypoK	165	156	220	13	

(h) hvCAS statistics	Sample	Spontaneous	Maximum Capture Frequency (Hz)						Spiral Induction
			N/A (capture at higher frequency)	1	1.5	2	2.5	3	
NormoK	0% (0/14)	1/14 (NC: 0.5, 1), (C: 1.5–3)	0/14	0/14	0/14	1/14	12/14	0% (0/14)
hypoK	21% (3/14)	5/14 (NC: 0.5–1.5 = 1), (NC: 0.5 = 3), (NC: 0.5–1 = 1)	0/14	0/14	0/14	3/14	6/14	50% (7/14)

NormoK, normokalaemia; hypoK, hypokalaemia; NC, non-capture; C, capture.

**TABLE 2:** hES2-hvCAS Calcium Transient (CaT) Parameters in acute hypokalaemia. Data analysis was performed for 1.5 Hz electrical stimulation recordings.

Parameters		Sample	Median	Lower 95% CI	Upper 95% CI	N	*p* Value
(a) Amplitude (ΔF represents the change in fluorescence intensity)		NormoK	0.861	0.707	1.16	12	*** < 0.001
		hypoK	1.37	1.28	1.5	13	
(b) CaT Duration (CaTD_x_) (Time duration in millisecond from the peak fluorescence intensity to the corresponding fluorescence decay intervals)	x						
	30%	NormoK	141	129	153	11	** = 0.001
		hypoK	161	150	170	10	
	50%	NormoK	223	213	235	11	** = 0.003
		hypoK	242	232	252	10	
	70%	NormoK	292	285	311	11	* = 0.009
		hypoK	318	306	330	10	
	90%	NormoK	379	358	392	11	** = 0.002
		hypoK	403	393	416	10	
(c) Time-to-peak/Activation Latency (Time duration in millisecond from the start of the rise of the fluorescence intensity to the peak fluorescence intensity)		NormoK	141	129	150	12	* = 0.045
		hypoK	153	143	158	11	
(d) Upstroke Velocity (ΔF/millisecond; ΔF represents the change in fluorescence intensity)		NormoK	0.00498	0.00456	0.00569	11	*** < 0.001
		hypoK	0.00801	0.0073	0.00923	10	

NormoK, normokalaemia; hypoK, hypokalaemia.

### Hypokalaemia Decreases Both Longitudinal and Transverse CVs of AP Without Altering the AR and Decreases ERPs

**FIGURE 5:**
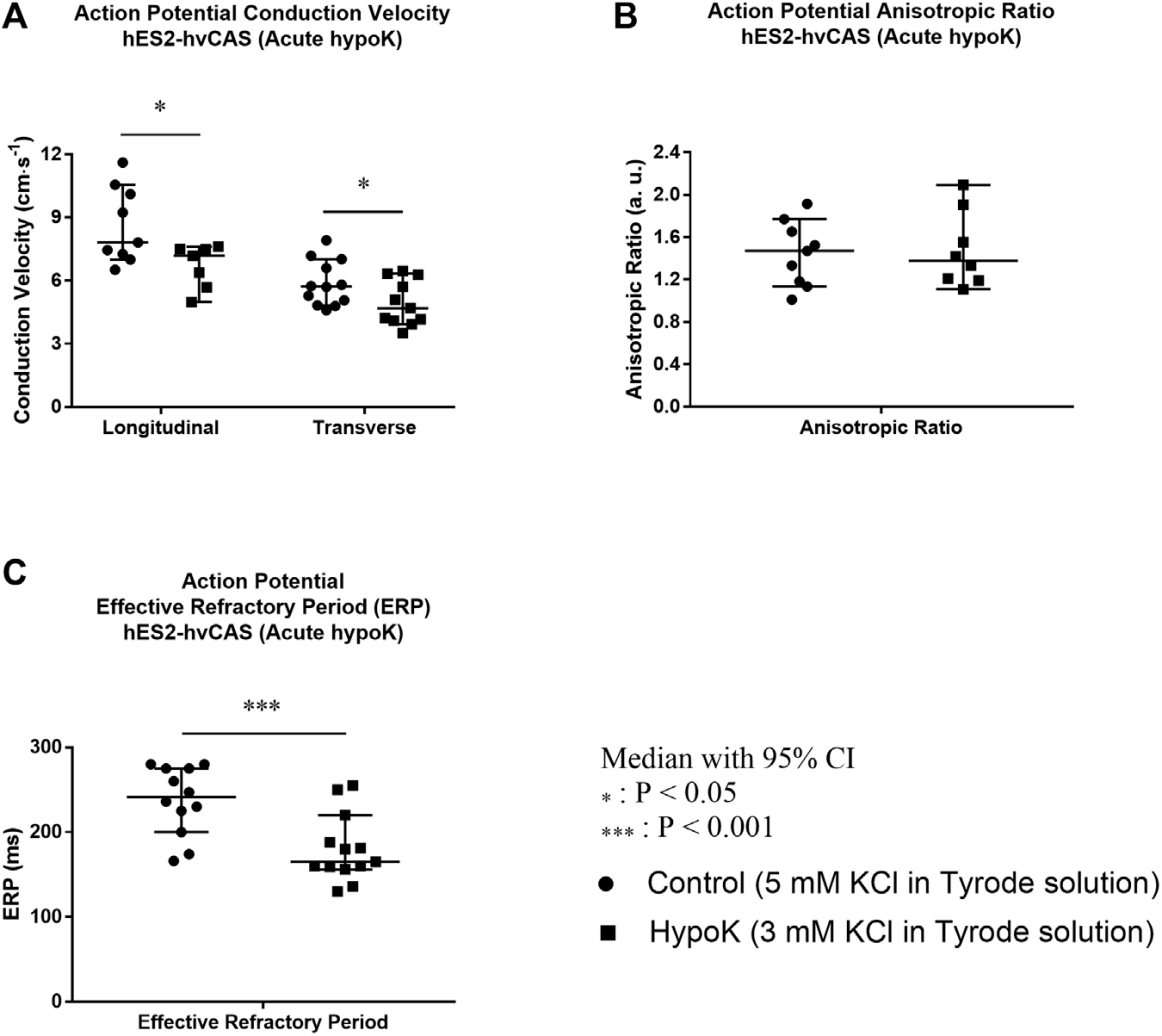
Human embryonic stem cell (hES2)-derived human ventricular cardiac anisotropic sheet (hvCAS) paced at 1.5 Hz during acute hypokalaemic (hypoK) experiment where the treatment was for 15 min before data acquisition. Number of samples is represented by n where the first number refers to control (5 mM KCl in Tyrode solution) samples and the second number refers to hypoK (3 mM KCl in Tyrode solution) samples. **(A)** Action Potential (AP) Longitudinal Conduction Velocity (LCV) (*n* = 9, 7)and Transverse Conduction Velocity (TCV) (*n* = 12, 11). The velocity was measured by calculating the speed of AP propagation from one point to the other. **(B)** AP Anisotropic Ratio (AR) which is the ratio of LCV to TCV. (*n* = 9, 8). **(C)** AP Effective Refractory Period (ERP). (*n* = 12, 13) Conduction Velocity decreased in hypokalaemic condition (asterisk, *p* < 0.05 for both LCV and TCV). ERP of hvCAS paced at 1.5 Hz (8 trains) followed by an extra premature stimulus which was gradually shortened to find ERP was decreased in hypokalaemic condition (asterisk, *p* < 0.001). AR is between 1.4 and 1.8, resembling more closer to the native human ventricle (AR = 3) in normokalaemia as well as in hypokalaemia.

There was a significant lowering of longitudinal CV of AP in hypokalaemia versus normokalaemia from 7.81 (7.01, 10.6) (n = 9) to 7.18 (4.99, 7.62) cm·s^−1^ (centimetre per second, n = 7; asterisk, *p* = 0.036; Figure 5A) and transverse CV of AP from 5.72 (4.83, 7.02) (n = 12) to 4.69 (3.93, 6.34) cm·s^−1^ (n = 11; asterisk, *p* = 0.030). However, the AR was not altered (normokalaemia: 1.47 (1.14, 1.77) (n = 9) vs. hypokalaemia: 1.38 (1.11, 2.09) a. u. (n = 8; ns, *p* = 0.444; Figure 5B). By contrast, ERPs were shortened from 242 (200, 275) (n = 12) to 165 (156, 220) ms significantly (n = 13; asterisks, *p* < 0.001; Figure 5C). Similar results were observed in chronic hypokalaemia for hES2-hvCAS as well as in hvCAS derived from N-iPSC (healthy control iPS cells) in both acute and chronic hypokalaemia except slight deviation. These results are summarized in Supplementary Tables SI–SVI).

Together all of the findings associate increased re-entrant arrhythmias observed in the form of spiral waves with depolarization, repolarization, and calcium handling abnormalities in the hypokalaemia model using our stem cell-derived bioengineered platform.

## Discussion

The main findings of this study are that hypokalaemia can induce arrhythmogenesis, which is associated with depolarization, repolarization, and calcium handling abnormalities in stem cell-derived bioengineered platforms. These confirm that hvCAS preparations can be used as a model system for studying human arrhythmic syndromes, providing opportunities for efficient drug screening programs for pro-arrhythmic effects.

Low extracellular K^+^ levels lead to a steeper transmembrane voltage gradient and membrane hyperpolarization (Tse et al., 2021). This low level also reduces the repolarization reserve by suppressing the rapid delayed rectifier (I_Kr_) channel, producing APD prolongation (Osadchii, 2010; Pezhouman et al., 2015). APD prolongation can in turn lead to the re-activation of L-type Ca^2+^ channels, causing EAD phenomena and triggering activity (Killeen et al., 2007). Moreover, it can predispose patients to re-entry through reduced CV, reduced tissue refractoriness, increased transmural dispersion of repolarization, and increased steepness of electrical restitution that can generate electrical alternans (Surawicz, 1985; Sabir et al., 2008; Tse et al., 2016a; Tse et al., 2016b). Reduction in CV is more likely due to APD prolongation. The membrane hyperpolarization also causes an increase in the availability of fast Na^+^ channels that leads to an increase in upstroke velocity (Osadchii, 2010). Despite the higher upstroke velocity, time-to-peak is prolonged, which is possibly due to higher amplitude caused by hyperpolarization. In our study, greater AP amplitudes, reduced CV, and longer APDs were observed in hvCAS. Moreover, both longitudinal and transverse conduction velocities were decreased to similar extents, leading to no significant alteration in the AR. This took a mean value between 1.4 and 1.8 in both under normokalaemic and hypokalaemic conditions, which is fairly close to the AR value for the human ventricle of 3 (Camelliti et al., 2011).

The amplitude and time-to-peak of CaTs were higher under hypokalaemic conditions. This can be explained by the suppression of sodium-calcium (Na^+^-Ca^2+^) exchanger and sodium-potassium pump during hypokalaemia, which leads to the accumulation of Ca^2+^ and Na^+^ in the cytoplasm (Pezhouman et al., 2015). Together with increased Ca^2+^ influx owing to L-type Ca^2+^ channel re-activation, this can lead to secondary depolarization events if the activation threshold is reached, an extra premature AP. The suppression of Na^+^-Ca^2+^ exchanger leads to the Ca^2+^ overload in the cytoplasm which in turn prolongs the time taken for Ca^2+^ to be removed from the cytosol via the following routes: Na^+^-Ca^2+^ exchanger to the extracellular space, sarco/endoplasmic reticulum Ca^2+^-ATPase, into the sarcoplasmic reticulum.

**FIGURE 6:**
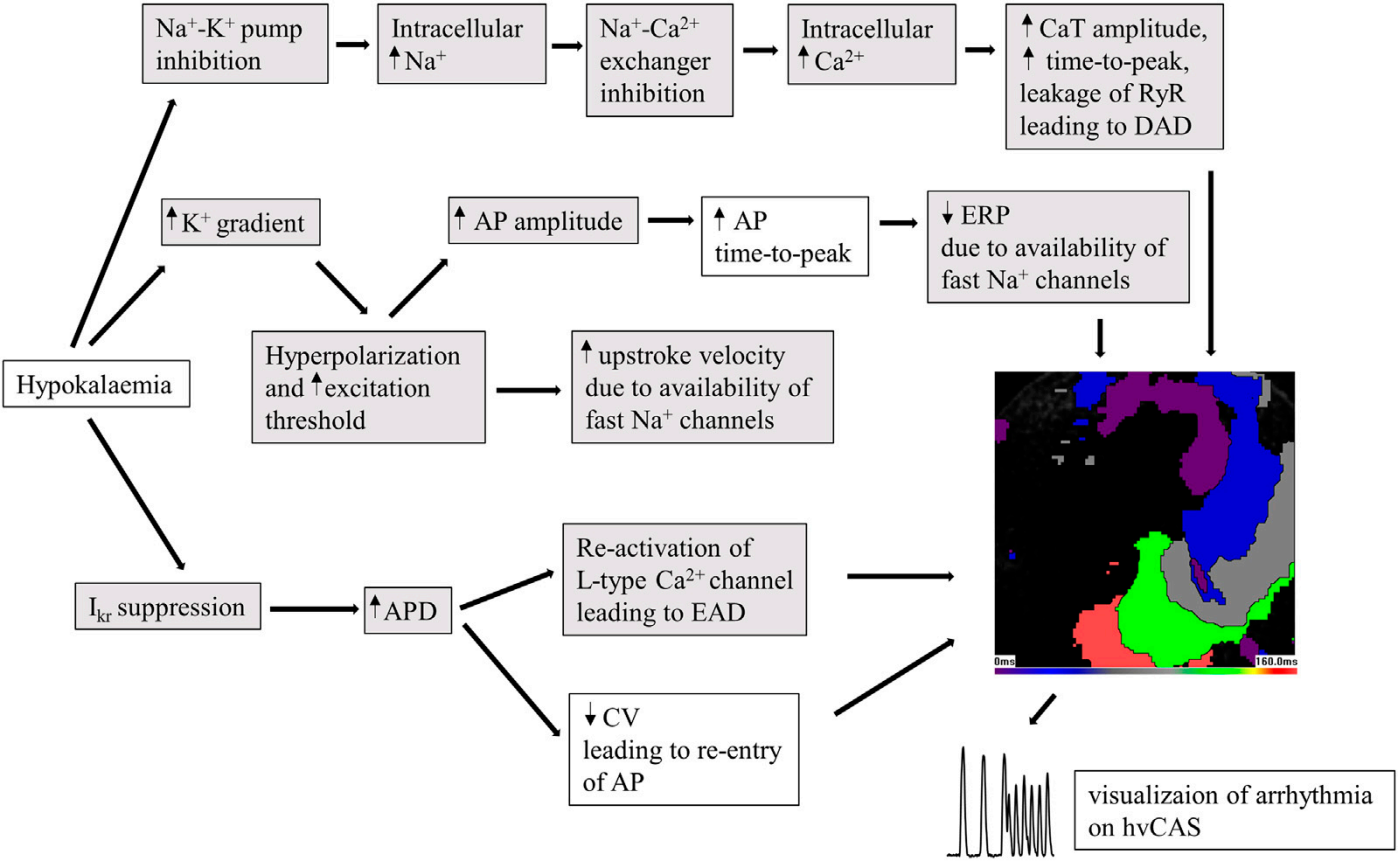
Diagram showing the changes caused by hypokalaemia in human pluripotent stem cell-derived human ventricular cardiac anisotropic sheet (hvCAS) during acute hypokalaemic (hypoK) experiment where the treatment was for 15 min before data acquisition. Hypokalaemia refers to 3 mM KCl in Tyrode solution on the samples. Those changes make cardiomyocytes prone to arrhythmia in hypokalaemic conditions. The boxes highlighted in grey are adapted from literature research. Those changes leading to arrhythmia can be visualized on the hvCAS.

The different electrophysiological mechanisms at play induced by hypokalaemia are summarized in Figure 6.

## Limitations of the Study

Although the cardiomyocytes (CMs) that we produce through directed cardiac differentiation are mostly ventricular CMs, there are still a small proportion of other subtypes of CMs (Weng et al., 2014). Additionally, stem cell-derived CMs show a relatively immature phenotype as reported in previous studies (Karakikes et al., 2015). By performing optical mapping on the hvCAS, relative values of voltage change and CaT can be obtained from the fluorescence intensity of the fast-responding voltage sensitive and Ca^2+^ dyes respectively. However, the fluorescence signal is not calibrated and absolute resting membrane potential cannot be determined. Furthermore, hvCAS contains a stack of cells at each pixel and the data presented here are from those multiple cells. This makes the AP upstroke phase appear slow compared to single-cell actual AP measured by patch-clamp or microelectrode array in other studies. Nevertheless, we can see the differences between normokalaemia and hypokalaemia hvCASs, and most importantly, arrhythmia can be visualized as spiral waves by using optical mapping, which is not possible by other techniques.

## Conclusion

The pro-arrhythmic effects of hypokalaemia are associated with depolarization, repolarization, and calcium handling abnormalities in a human multicellular monolayer model using a stem cell-derived bioengineered platform. This platform can be used as a model system for cardiac arrhythmic risk assessment where arrhythmia can be visualized as spiral waves.

## Data Availability

The original contributions presented in the study are included in the article/Supplementary Materials, further inquiries can be directed to the corresponding authors.
