# Supplementary material for: Arrhythmic Risk Assessment of Hypokalaemia Using Human Pluripotent Stem Cell-Derived Cardiac Anisotropic Sheets
Source: Front Cell Dev Biol. 2021 Dec 6;9:681665. doi: 10.3389/fcell.2021.681665 (PMC8685904; doi:10.3389/fcell.2021.681665)
Supplement: Supplementary file 1 [file Presentation1.PPTX]

## Slide 1
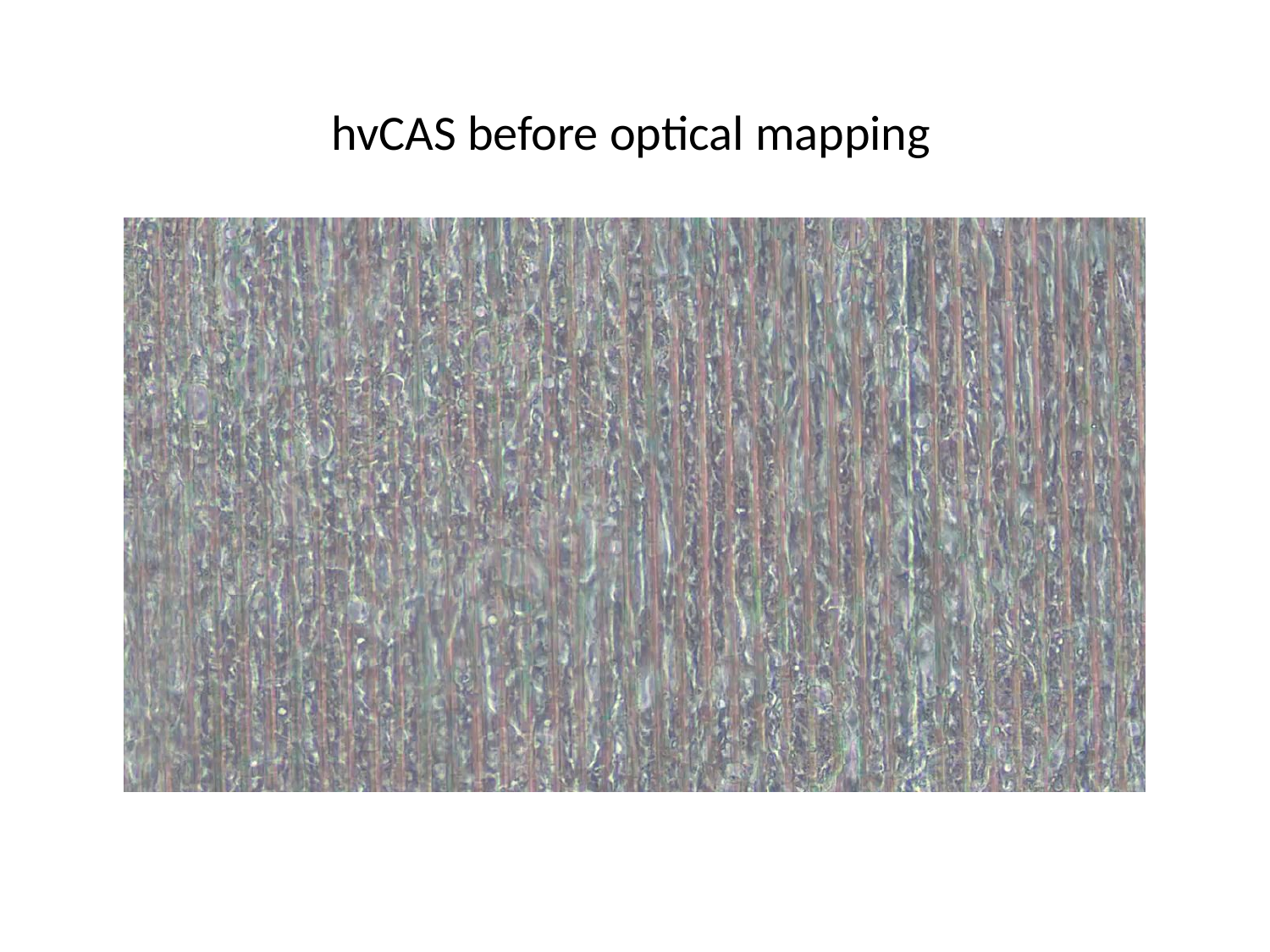

hvCAS before optical mapping

## Slide 2
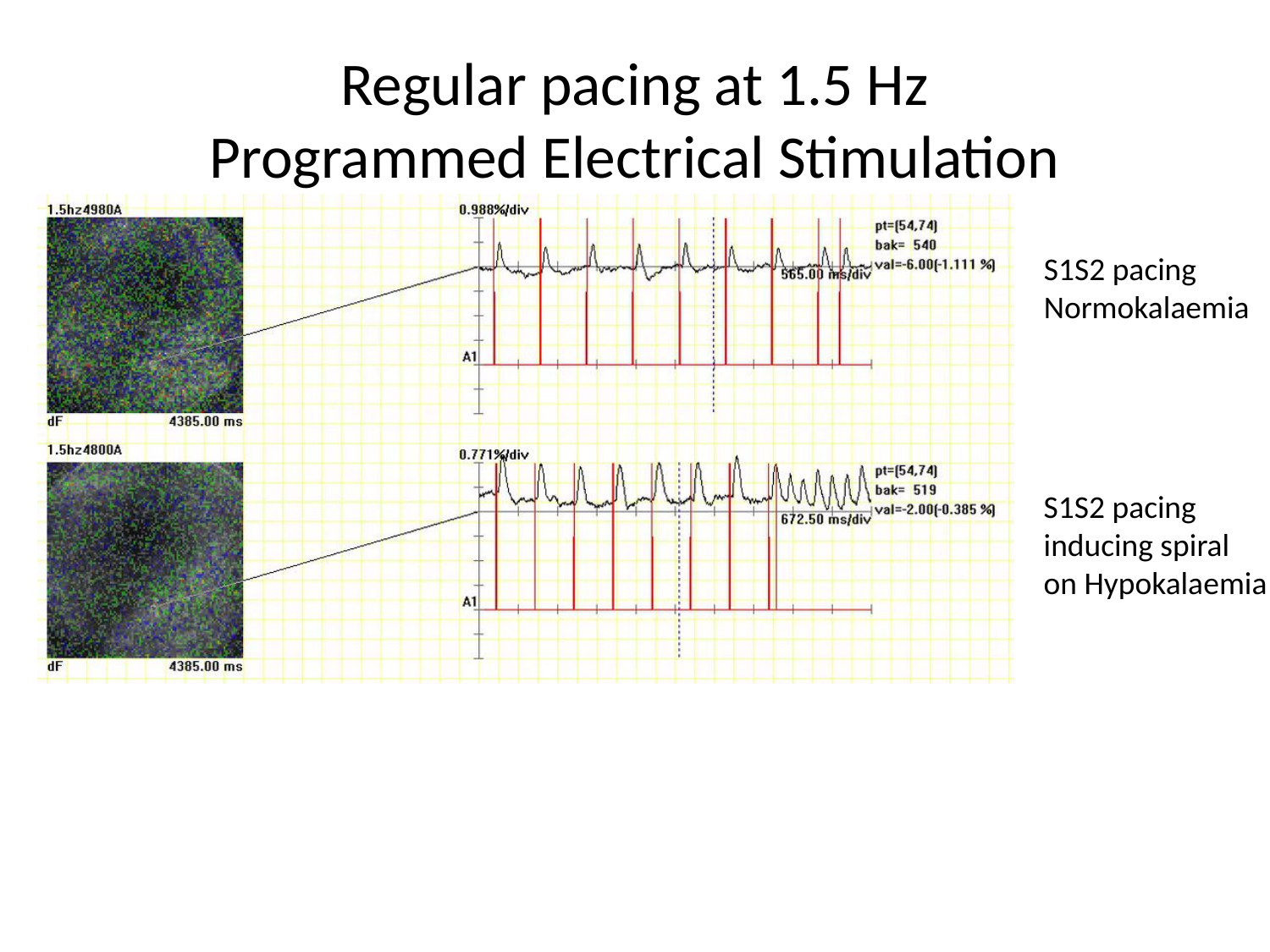

Regular pacing at 1.5 Hz
Programmed Electrical Stimulation
S1S2 pacing
Normokalaemia
S1S2 pacing
inducing spiral
on Hypokalaemia

## Slide 3
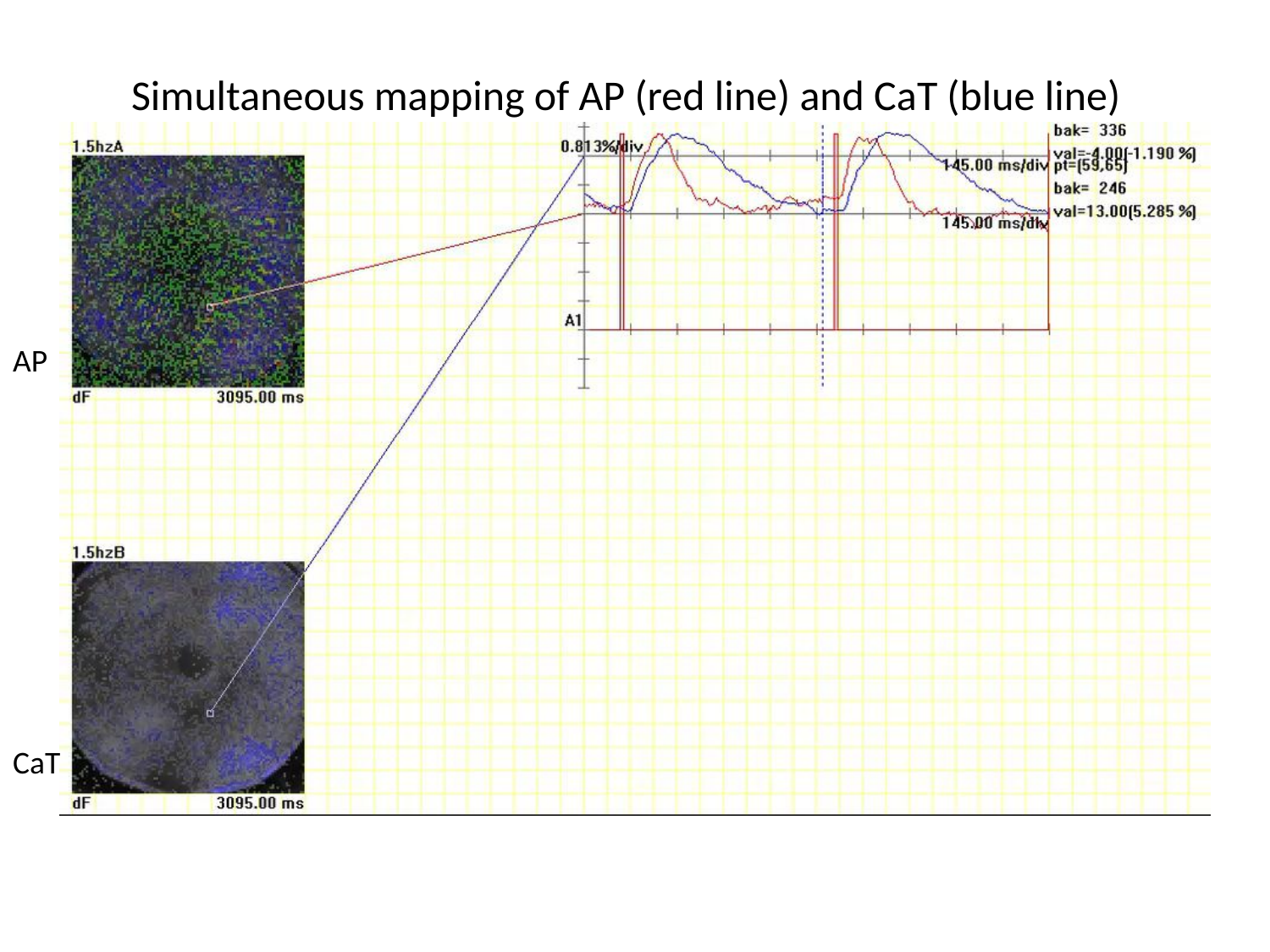

Simultaneous mapping of AP (red line) and CaT (blue line)
AP
CaT
