# Supplementary material for: Arrhythmic Risk Assessment of Hypokalaemia Using Human Pluripotent Stem Cell-Derived Cardiac Anisotropic Sheets
Source: Front Cell Dev Biol. 2021 Dec 6;9:681665. doi: 10.3389/fcell.2021.681665 (PMC8685904; doi:10.3389/fcell.2021.681665)
Supplement: Supplementary file 2 [file DataSheet1.doc]

Supplementary Material

# Supplementary Tables

**Table I) hES2-hvCAS Action Potential (AP) Parameters in chronic hypokalaemia. Data analysis was performed for 1.5 Hz electrical stimulation recordings.**

| Parameters | | | | | Sample | Median | | Lower 95% CI | | | Upper 95% CI | | | N | P value |
| --- | --- | --- | --- | --- | --- | --- | --- | --- | --- | --- | --- | --- | --- | --- | --- |
| 1. Amplitude (ΔF represents the change in fluorescence intensity). | | | | | NormoK | 0.652 | | 0.609 | | | 0.708 | | | 3 | - ***   < 0.001 |
|  |  |  |  |  | hypoK | 0.939 | | 0.829 | | | 1.06 | | | 5 |  |
| 1. AP Duration (APD_x_) (Time duration in millisecond from the peak fluorescence intensity to the corresponding fluorescence decay intervals). | | | x | |  | | | | | | | | | | |
|  |  |  | 30% | | NormoK | 38.9 | | 33.6 | | | 42.3 | | | 3 | - *   = 0.006 |
|  |  |  |  |  | hypoK | 44.8 | | 40.2 | | | 49.3 | | | 5 |  |
|  |  |  | 50% | | NormoK | 54.8 | | 50.9 | | | 58.4 | | | 3 | - ***   < 0.001 |
|  |  |  |  |  | hypoK | 63.4 | | 58.3 | | | 74.8 | | | 5 |  |
|  |  |  | 70% | | NormoK | 72.3 | | 68.1 | | | 82.6 | | | 3 | - ***   < 0.001 |
|  |  |  |  |  | hypoK | 87.5 | | 78.2 | | | 99.9 | | | 5 |  |
|  |  |  | 90% | | NormoK | 102 | | 86.2 | | | 110 | | | 3 | - ***   < 0.001 |
|  |  |  |  |  | hypoK | 128 | | 105 | | | 141 | | | 5 |  |
| 1. Time-to-peak / Activation Latency (Time duration in millisecond from the start of the rise of the fluorescence intensity to the peak fluorescence intensity). | | | | | NormoK | 82.8 | | 77.8 | | | 91.5 | | | 3 | - ns   = 0.306 |
|  |  |  |  |  | hypoK | 84.1 | | 79.6 | | | 94.1 | | | 5 |  |
| 1. Upstroke Velocity (ΔF/millisecond; ΔF represents the change in fluorescence intensity). | | | | | NormoK | 0.007 | | 0.00652 | | | 0.00757 | | | 3 | - ***   < 0.001 |
|  |  |  |  |  | hypoK | 0.00842 | | 0.00778 | | | 0.0132 | | | 5 |  |
| 1. Conduction Velocity (CV) (cm·s^-1^; AP propagation between two points). | | | Longitudinal (L) CV | | NormoK | 6.41 | | 5.83 | | | 7.48 | | | 3 | - ns   = 0.400 |
|  |  |  |  |  | hypoK | 5.79 | | 4.13 | | | 7.45 | | | 2 |  |
|  |  |  | Transverse (T) CV | | NormoK | 3.73 | | 3.52 | | | 5.14 | | | 3 | - ns   = 0.200 |
|  |  |  |  |  | hypoK | 3.17 | | 2.82 | | | 3.52 | | | 2 |  |
| 1. Anisotropic Ratio (Ratio of LCV to TCV). | | | | | NormoK | 1.66 | | 1.46 | | | 1.72 | | | 3 | - ns   = 0.400 |
|  |  |  |  |  | hypoK | 1.79 | | 1.47 | | | 2.12 | | | 2 |  |
| 1. Effective Refractory Period (millisecond). | | | | | NormoK | 340 | | 279 | | | 430 | | | 7 | - *   = 0.035 |
|  |  |  |  |  | hypoK | 278 | | 241 | | | 309 | | | 5 |  |
| 1. hvCAS statistics. | Sample | Spontaneous | | Maximum Capture Frequency (Hz.) | | | | | | | | | | | Spiral Induction |
|  |  |  |  | - N/A (capture at higher frequency) | | | - 1 | | - 1.5 | - 2 | | - 2.5 | - 3 | |  |
|  | NormoK | - 0% - (0/8) | | - 0/8 | | | - 0/8 | | - 1/8 | - 1/8 | | - 3/8 | - 3/8 | | 12.5%   - (1/8) |
|  | hypoK | - 0% - (0/6) | | - 0/6 | | | - 0/6 | | - 0/6 | - 0/6 | | - 1/6 | - 5/6 | | 33.3%   - (2/6) |

**Note: NormoK = normokalaemia, hypoK = hypokalaemia, NC = non-capture, C = capture**

**Table II) hES2-hvCAS Calcium Transient (CaT) Parameters in chronic hypokalaemia. Data analysis was performed for 1.5 Hz electrical stimulation recordings.**

| Parameters | | Sample | Median | Lower 95% CI | Upper 95% CI | N | P value |
| --- | --- | --- | --- | --- | --- | --- | --- |
| 1. Amplitude (ΔF represents the change in fluorescence intensity). | | NormoK | 1.68 | 1.33 | 2.16 | 4 | - **   = 0.001 |
|  |  | hypoK | 2.49 | 1.75 | 3.11 | 7 |  |
| 1. CaT Duration (CaTD_x_) (Time duration in millisecond from the peak fluorescence intensity to the corresponding fluorescence decay intervals). | x |  | | | | |  |
|  | 30% | NormoK | 128 | 117 | 149 | 4 | - **   = 0.002 |
|  |  | hypoK | 160 | 140 | 181 | 7 |  |
|  | 50% | NormoK | 220 | 195 | 239 | 4 | - *   = 0.015 |
|  |  | hypoK | 233 | 212 | 262 | 7 |  |
|  | 70% | NormoK | 321 | 277 | 337 | 4 | - ns   = 0.142 |
|  |  | hypoK | 296 | 269 | 317 | 5 |  |
|  | 90% | NormoK | 410 | 383 | 434 | 4 | - *   = 0.037 |
|  |  | hypoK | 380 | 348 | 399 | 5 |  |
| 1. Time-to-peak / Activation Latency (Time duration in millisecond from the start of the rise of the fluorescence intensity to the peak fluorescence intensity). | | NormoK | 126 | 118 | 139 | 4 | - ***   < 0.001 |
|  |  | hypoK | 154 | 140 | 169 | 7 |  |
| 1. Upstroke Velocity (ΔF/millisecond; ΔF represents the change in fluorescence intensity). | | NormoK | 0.0116 | 0.0107 | 0.0138 | 4 | - ns   = 0.32 |
|  |  | hypoK | 0.0112 | 0.00998 | 0.0136 | 7 |  |

**Note: NormoK = normokalaemia, hypoK = hypokalaemia**

**Table III) N-iPSC-hvCAS Action Potential (AP) Parameters in acute hypokalaemia. Data analysis was performed for 1.5 Hz electrical stimulation recordings.**

| Parameters | | | | | Sample | Median | | Lower 95% CI | | | Upper 95% CI | | | N | P value |
| --- | --- | --- | --- | --- | --- | --- | --- | --- | --- | --- | --- | --- | --- | --- | --- |
| 1. Amplitude (ΔF represents the change in fluorescence intensity). | | | | | NormoK | 1.54 | | 1.44 | | | 1.64 | | | 29 | - **   = 0.001 |
|  |  |  |  |  | hypoK | 1.69 | | 1.64 | | | 1.79 | | | 28 |  |
| 1. AP Duration (APD_x_) (Time duration in millisecond from the peak fluorescence intensity to the corresponding fluorescence decay intervals). | | | x | |  | | | | | | | | | | |
|  |  |  | 30% | | NormoK | 48.8 | | 45 | | | 53.5 | | | 29 | - ns   = 0.197 |
|  |  |  |  |  | hypoK | 44.4 | | 39.9 | | | 48.9 | | | 28 |  |
|  |  |  | 50% | | NormoK | 67.6 | | 62.7 | | | 73 | | | 29 | - ns   = 0.238 |
|  |  |  |  |  | hypoK | 61.9 | | 57.4 | | | 67.6 | | | 28 |  |
|  |  |  | 70% | | NormoK | 89.5 | | 85 | | | 94.5 | | | 29 | - ns   = 0.167 |
|  |  |  |  |  | hypoK | 83 | | 77.7 | | | 88.4 | | | 28 |  |
|  |  |  | 90% | | NormoK | 118 | | 109 | | | 123 | | | 29 | - ns   = 0.442 |
|  |  |  |  |  | hypoK | 115 | | 110 | | | 123 | | | 28 |  |
| 1. Time-to-peak / Activation Latency (Time duration in millisecond from the start of the rise of the fluorescence intensity to the peak fluorescence intensity). | | | | | NormoK | 91.6 | | 87.9 | | | 95.5 | | | 29 | - ns   = 0.195 |
|  |  |  |  |  | hypoK | 88.2 | | 85.5 | | | 90.9 | | | 28 |  |
| 1. Upstroke Velocity (ΔF/millisecond; ΔF represents the change in fluorescence intensity). | | | | | NormoK | 0.0168 | | 0.0163 | | | 0.0176 | | | 29 | - *   = 0.012 |
|  |  |  |  |  | hypoK | 0.0171 | | 0.0166 | | | 0.0178 | | | 28 |  |
| 1. Conduction Velocity (CV) (cm·s^-1^; AP propagation between two points). | | | Longitudinal (L) CV | | NormoK | 12.2 | | 9.97 | | | 16.1 | | | 18 | - *   = 0.03 |
|  |  |  |  |  | hypoK | 10.9 | | 7.65 | | | 12.9 | | | 18 |  |
|  |  |  | Transverse (T) CV | | NormoK | 7.78 | | 7.18 | | | 9.55 | | | 15 | - ***   < 0.001 |
|  |  |  |  |  | hypoK | 6.28 | | 5.14 | | | 7.31 | | | 14 |  |
| 1. Anisotropic Ratio (Ratio of LCV to TCV). | | | | | NormoK | 1.96 | | 1.3 | | | 2.08 | | | 15 | - ns   = 0.295 |
|  |  |  |  |  | hypoK | 1.63 | | 1.26 | | | 2.07 | | | 14 |  |
| 1. Effective Refractory Period (millisecond). | | | | | NormoK | 280 | | 250 | | | 310 | | | 21 | - *   = 0.046 |
|  |  |  |  |  | hypoK | 248 | | 210 | | | 287 | | | 18 |  |
| 1. hvCAS statistics. | Sample | Spontaneous | | Maximum Capture Frequency (Hz.) | | | | | | | | | | | Spiral Induction |
|  |  |  |  | - N/A (capture at higher frequency) | | | - 1 | | - 1.5 | - 2 | | - 2.5 | - 3 | |  |
|  | NormoK | - 0% - (0/29) | | - 1/29 - (NC: 0.5, 1), - (C: 1.5-3) | | | - 4/29 | | - 2/29 | - 2/29 | | - 5/29 | - 15/29 | | 3.4%   - (1/29) |
|  | hypoK | - 3.6% - (1/28) | | - 7/28 - (NC: 0.5-2.5 = 1), (NC: 0.5 = 6) | | | - 3/28 | | - 3/28 | - 2/28 | | - 2/28 | - 11/28 | | 10.7%   - (3/28) |

**Note: NormoK = normokalaemia, hypoK = hypokalaemia, NC = non-capture, C = capture**

**Table IV) N-iPSC-hvCAS Calcium Transient (CaT) Parameters in acute hypokalaemia. Data analysis was performed for 1.5 Hz electrical stimulation recordings.**

| Parameters | | Sample | Median | Lower 95% CI | Upper 95% CI | N | P value |
| --- | --- | --- | --- | --- | --- | --- | --- |
| 1. Amplitude (ΔF represents the change in fluorescence intensity). | | NormoK | 1.5 | 1.34 | 1.71 | 18 | - ***   < 0.001 |
|  |  | hypoK | 3.28 | 2.8 | 4.21 | 24 |  |
| 1. CaT Duration (CaTD_x_) (Time duration in millisecond from the peak fluorescence intensity to the corresponding fluorescence decay intervals). | x |  | | | | |  |
|  | 30% | NormoK | 140 | 136 | 143 | 25 | - ns   = 0.062 |
|  |  | hypoK | 136 | 131 | 141 | 24 |  |
|  | 50% | NormoK | 211 | 206 | 216 | 25 | - *   = 0.010 |
|  |  | hypoK | 204 | 198 | 209 | 24 |  |
|  | 70% | NormoK | 286 | 281 | 291 | 25 | - ns   = 0.086 |
|  |  | hypoK | 281 | 272 | 289 | 24 |  |
|  | 90% | NormoK | 359.8 | 354 | 368 | 25 | - *   = 0.046 |
|  |  | hypoK | 370 | 364 | 377 | 24 |  |
| 1. Time-to-peak / Activation Latency (Time duration in millisecond from the start of the rise of the fluorescence intensity to the peak fluorescence intensity). | | NormoK | 157.3 | 153 | 161 | 25 | - ns   = 0.122 |
|  |  | hypoK | 158.1 | 153 | 164 | 24 |  |
| 1. Upstroke Velocity (ΔF/millisecond; ΔF represents the change in fluorescence intensity). | | NormoK | 0.007871 | 0.007 | 0.00934 | 20 | - ***   < 0.001 |
|  |  | hypoK | 0.01623 | 0.0142 | 0.0213 | 24 |  |

**Note: NormoK = normokalaemia, hypoK = hypokalaemia**

**Table V) N-iPSC-hvCAS Action Potential (AP) Parameters in chronic hypokalaemia. Data analysis was performed for 1.5 Hz electrical stimulation recordings.**

| Parameters | | | | | Sample | Median | | Lower 95% CI | | | Upper 95% CI | | | N | P value |
| --- | --- | --- | --- | --- | --- | --- | --- | --- | --- | --- | --- | --- | --- | --- | --- |
| 1. Amplitude (ΔF represents the change in fluorescence intensity). | | | | | NormoK | 1.279 | | 1.2 | | | 1.34 | | | 33 | - ***   < 0.001 |
|  |  |  |  |  | hypoK | 1.522 | | 1.44 | | | 1.59 | | | 29 |  |
| 1. AP Duration (APD_x_) (Time duration in millisecond from the peak fluorescence intensity to the corresponding fluorescence decay intervals). | | | x | |  | | | | | | | | | |  |
|  |  |  | 30% | | NormoK | 42.44 | | 40.6 | | | 44 | | | 33 | - ***   < 0.001 |
|  |  |  |  |  | hypoK | 48.83 | | 46.4 | | | 51.1 | | | 29 |  |
|  |  |  | 50% | | NormoK | 60.35 | | 58.1 | | | 62.2 | | | 33 | - ***   < 0.001 |
|  |  |  |  |  | hypoK | 68.01 | | 65.4 | | | 72.1 | | | 29 |  |
|  |  |  | 70% | | NormoK | 80.88 | | 78.2 | | | 83.7 | | | 33 | - ***   < 0.001 |
|  |  |  |  |  | hypoK | 92.25 | | 89.2 | | | 95.5 | | | 29 |  |
|  |  |  | 90% | | NormoK | 109.5 | | 106 | | | 113 | | | 33 | - ***   < 0.001 |
|  |  |  |  |  | hypoK | 126.1 | | 122 | | | 132 | | | 29 |  |
| 1. Time-to-peak / Activation Latency (Time duration in millisecond from the start of the rise of the fluorescence intensity to the peak fluorescence intensity). | | | | | NormoK | 82.46 | | 80.6 | | | 84.6 | | | 33 | - ***   < 0.001 |
|  |  |  |  |  | hypoK | 93.87 | | 89.3 | | | 95.8 | | | 29 |  |
| 1. Upstroke Velocity (ΔF/millisecond; ΔF represents the change in fluorescence intensity). | | | | | NormoK | 0.01259 | | 0.0122 | | | 0.0134 | | | 33 | - ***   < 0.001 |
|  |  |  |  |  | hypoK | 0.01512 | | 0.0141 | | | 0.0156 | | | 29 |  |
| 1. Conduction Velocity (CV) (cm·s^-1^; AP propagation between two points). | | | Longitudinal (L) CV | | NormoK | 9.106 | | 8.45 | | | 10.6 | | | 30 | - *   = 0.005 |
|  |  |  |  |  | hypoK | 7.919 | | 7.19 | | | 9.21 | | | 27 |  |
|  |  |  | Transverse (T) CV | | NormoK | 6.65 | | 5.8 | | | 8.04 | | | 28 | - *   = 0.026 |
|  |  |  |  |  | hypoK | 6.04 | | 4.95 | | | 6.8 | | | 25 |  |
| 1. Anisotropic Ratio (Ratio of LCV to TCV). | | | | | NormoK | 1.478 | | 1.27 | | | 1.6 | | | 27 | - ns   = 0.213 |
|  |  |  |  |  | hypoK | 1.339 | | 1.06 | | | 1.67 | | | 24 |  |
| 1. Effective Refractory Period (millisecond). | | | | | NormoK | 242 | | 221 | | | 259 | | | 18 | - *   = 0.018 |
|  |  |  |  |  | hypoK | 218 | | 201 | | | 244 | | | 14 |  |
| 1. hvCAS statistics. | Sample | Spontaneous | | Maximum Capture Frequency (Hz.) | | | | | | | | | | | Spiral Induction |
|  |  |  |  | - N/A (capture at higher frequency) | | | - 1 | | - 1.5 | - 2 | | - 2.5 | - 3 | |  |
|  | NormoK | - 30.3% - (10/33) | | - 4/33 - (NC: 0.5, 1, 2 = 1), (NC: 0.5 = 2) - (NC: 0.5, 1 = 1) | | | - 1/33 | | - 3/33 | - 1/33 | | - 3/33 | - 21/33 | | 3.0%   - (1/33) |
|  | hypoK | - 38.7% - (12/31) | | - 7/31 - (NC: 0.5 = 3), (NC: 0.5, 1 = 1) - (NC: 0.5-1.5 = 1), - (NC: 0.5, 1.5-3 = 1), - (NC: 0.5, 1, 2.5, 3 = 1) | | | - 5/31 | | - 0/31 | - 0/31 | | - 5/31 | - 14/31 | | 0%   - (0/31) |

**Note: NormoK = normokalaemia, hypoK = hypokalaemia, NC = non-capture, C = capture**

**Table VI) N-iPSC-hvCAS Calcium Transient (CaT) Parameters in chronic hypokalaemia. Data analysis was performed for 1.5 Hz electrical stimulation recordings.**

| Parameters | | Sample | Median | Lower 95% CI | Upper 95% CI | N | P value |
| --- | --- | --- | --- | --- | --- | --- | --- |
| 1. Amplitude (ΔF represents the change in fluorescence intensity). | | NormoK | 1.168 | 1.09 | 1.23 | 8 | - ***   < 0.001 |
|  |  | hypoK | 2.019 | 1.88 | 2.3 | 13 |  |
| 1. CaT Duration (CaTD_x_) (Time duration in millisecond from the peak fluorescence intensity to the corresponding fluorescence decay intervals). | x |  | | | | |  |
|  | 30% | NormoK | 133 | 123 | 142 | 10 | - ***   < 0.001 |
|  |  | hypoK | 144 | 139 | 150 | 14 |  |
|  | 50% | NormoK | 201 | 190 | 206 | 10 | - ***   < 0.001 |
|  |  | hypoK | 216 | 208 | 225 | 14 |  |
|  | 70% | NormoK | 272 | 266 | 280 | 10 | - ***   < 0.001 |
|  |  | hypoK | 291 | 285 | 299 | 14 |  |
|  | 90% | NormoK | 361 | 350 | 368 | 10 | - ***   < 0.001 |
|  |  | hypoK | 382.5 | 375 | 387 | 14 |  |
| 1. Time-to-peak / Activation Latency (Time duration in millisecond from the start of the rise of the fluorescence intensity to the peak fluorescence intensity). | | NormoK | 130.2 | 125 | 136 | 10 | - ***   < 0.001 |
|  |  | hypoK | 152.3 | 147 | 158 | 14 |  |
| 1. Upstroke Velocity (ΔF/millisecond; ΔF represents the change in fluorescence intensity). | | NormoK | 0.00792 | 0.00725 | 0.00837 | 8 | - ***   < 0.001 |
|  |  | hypoK | 0.0105 | 0.00987 | 0.0119 | 12 |  |

**Note: NormoK = normokalaemia, hypoK = hypokalaemia**


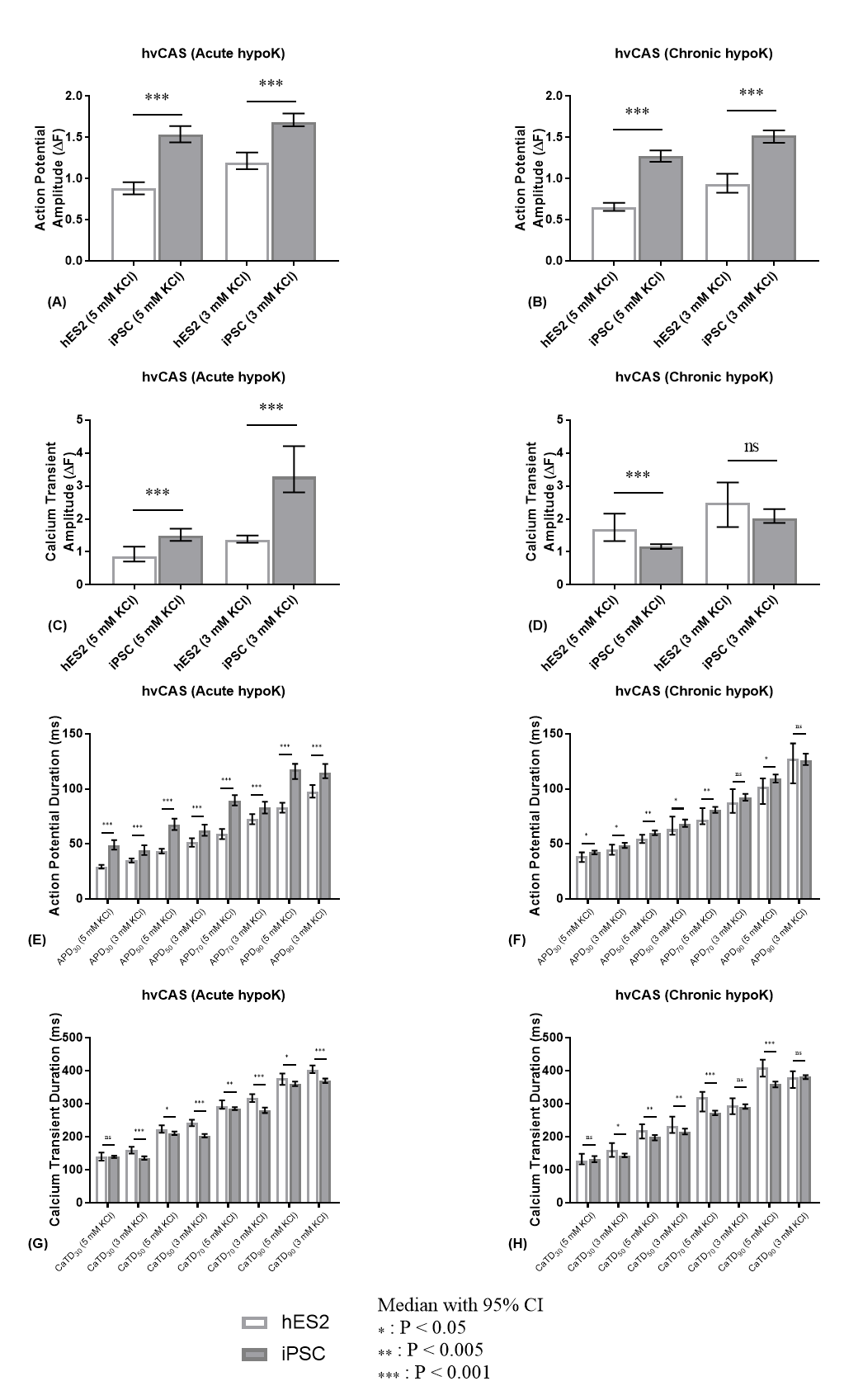


**Figure 1: Comparison of hES2- and iPSC- hvCAS in normokalaemia, and acute and chronic hypokalaemia condition.**

**Parameters compared: Action Potential Amplitude, Calcium Transient Amplitude, Action Potential Duration, Calcium Transient Duration**


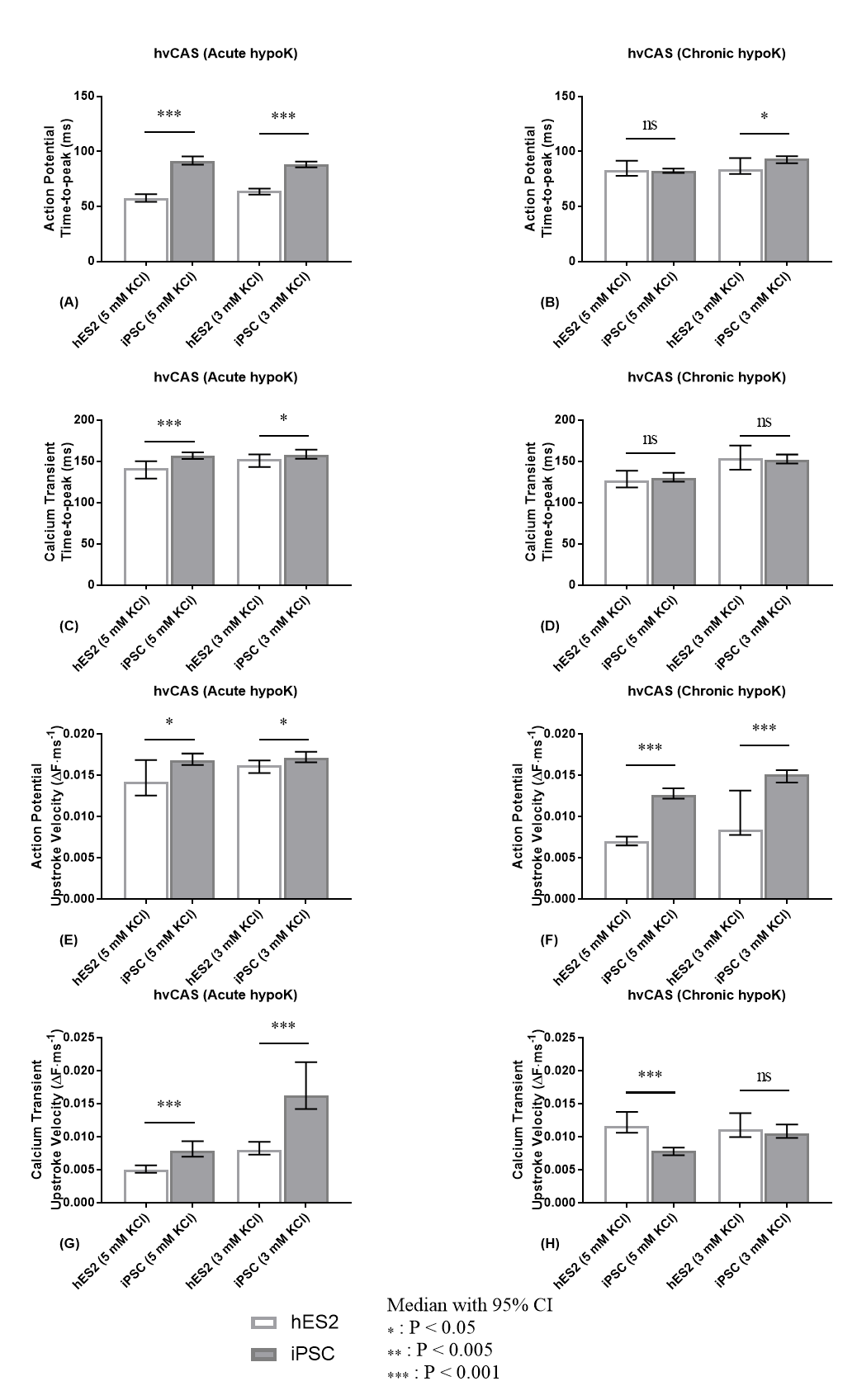


**Figure 2: Comparison of hES2- and iPSC- hvCAS in normokalaemia, and acute and chronic hypokalaemia condition.**

**Parameters compared: Action Potential Time-to-peak, Calcium Transient Time-to-peak, Action Potential Upstroke velocity, Calcium Transient Upstroke velocity**


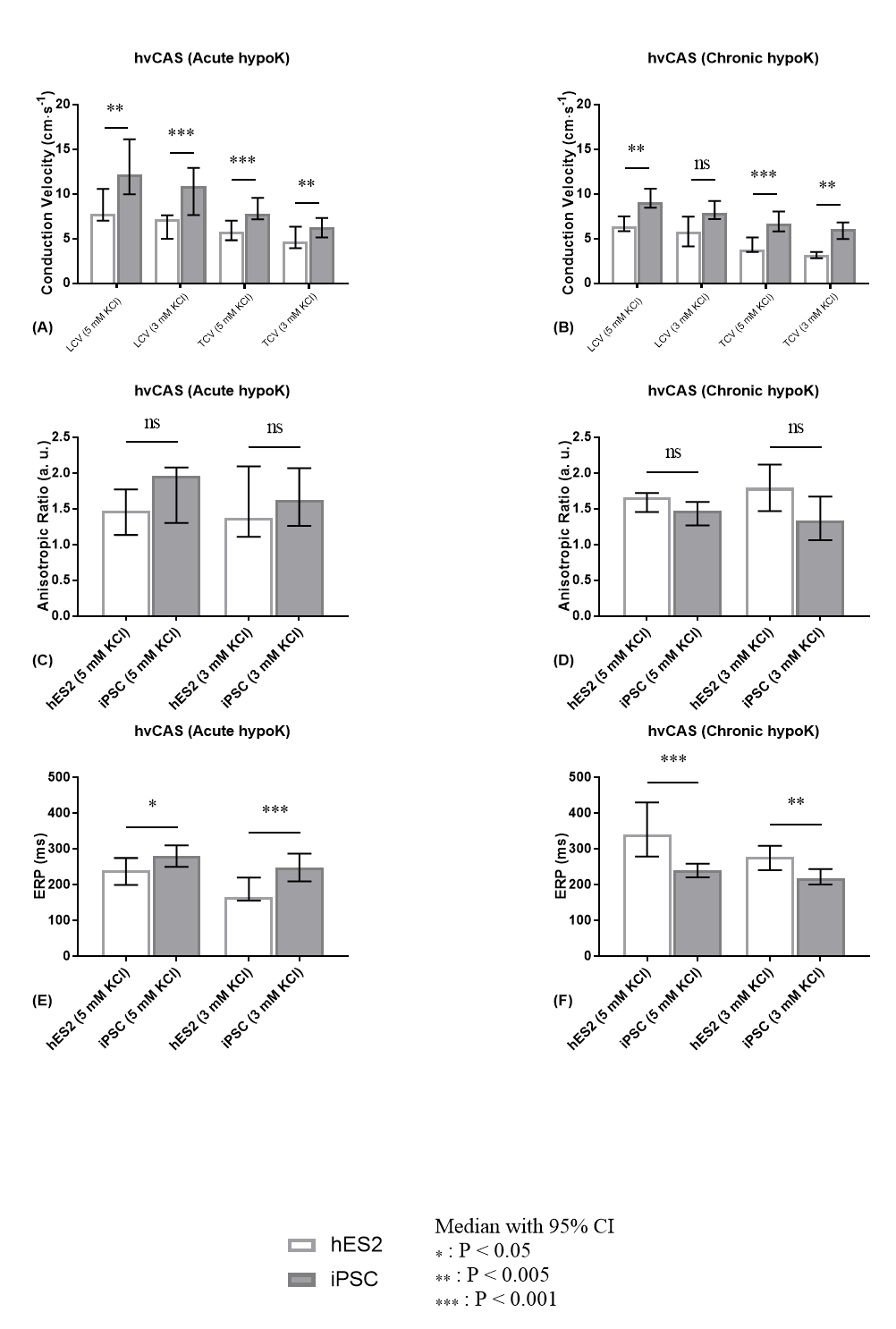


**Figure 3: Comparison of hES2- and iPSC- hvCAS in normokalaemia, and acute and chronic hypokalaemia condition.**

**Parameters compared: Conduction velocity, Anisotropic ratio, Effective refractory period**
